# Military Medical Notes

**Published:** 1916-04-01

**Authors:** 


### MILITARY MEDICAL NOTES.

The rumour of Sir Alfred Keogh's resignation of the office of Director-General of the Army Medical Services had, even for these rapid days, a very brief existence. Improbable in itself, it became ridiculous when it was associated with the complaints which had been made relative to the failure of the medical equipment of the Forces now engaged in Mesopotamia. Whoever was responsible for this defect, it certainly was not the Director-General, as the organisation of the expedition was undertaken, not by the Home Government but by the Government of India. Hence it was absurd to suppose that Sir Alfred Keogh would in the crisis of a great war withdraw from a duty for which everyone agrees he is admirably suited, merely because there were imperfect medical arrangements in a department over which he exercised no control. As most people know, Sir Alfred Keogh had completed prior to the outbreak of the war a term of service as Director-General, and had after his retiral accepted the position of Rector of the Imperial College of Science and Technology. He was recalled to the War Office by the national emergency, and thus it has come about that he is at present directing an organisation which in an earlier day he did much to establish on sound lines. Colonel Lee, at the commencement of the war, was appointed the special personal representative of Lord Kitchener to report upon the practical work of the Medical Services in the field. In his speech in the House he vigorously refuted the charges which had been advanced by Mr. McNeill, and justified the medical practitioners serving with the Forces as a highly skilled and gallant body of men, who deserved not carping criticism, but the whole-hearted gratitude of the entire community. In lighter vein he reminded the House that the present critic of the doctors was the same gentleman who in earlier days had discovered some two millions of anticipated "war-babies," and that the sequel had reduced these to two false alarms and a case of twins!

### Sir A. Keogh's Assistant.

Surgeon-General W. Babtie, V.C., who has been appointed to assist the Director-General, has had a very distinguished military career, and is likely to go in due course to a still higher position. His name became known to the public in the Boer War, when at the Battle of Colenso he distinguished himself by attending to the wounded under fire and by going to the rescue of Lord Roberts' son, who had been mortally wounded. It was for this he was decorated with the V.C. At an earlier date he had received the C.M.G. for services in Crete, and more recently he has held high office in India. His record and his earlier service at the War Office under Sir Alfred Keogh guarantee the success of the new arrangement, and the Army will not fail to profit from it. Surgeon-General Babtie is a medical graduate of the University of Glasgow. The Director-General has dealt energetically with the statement that there is no further need for increasing the numbers of medical officers serving with the Army. This statement when it appeared in print was credited to the Director-General himself. But in a letter to the Secretary of the Irish Medical War Committee, Sir Alfred Keogh describes it as "entirely false," and as "very far from anything I have ever said." He adds that merely to meet the war wastage in France he needs 150 new medical officers every month, and it must be presumed that further numbers are needed for the increasing armies now being formed. So precise an announcement is a great advantage to everyone concerned, and it has done much to clear the air. Rightly or wrongly, an impression had been created that the Army had a quite sufficient number of doctors, and that the call for more was inspired rather by the Central War Committee than by the War Office. Now that the necessities are known, the work of this Committee will be greatly facilitated, for it is impossible to believe that the profession will allow the Army to be ill-supplied with medical officers. The Hospital has already advocated greater publicity in the proceedings of the Committee. It is a very serious thing to ask practitioners to leave their practices, and the request ought to be accompanied by exact information as to the necessities of the position. General statements, whether by the War Office or by a professional committee, are hardly adequate to the occasion. But when definite figures are produced, there can be no doubt the necessary doctors will be forthcoming.

### A Genuine Difficulty.

The greatest difficulty in persuading practitioners to enrol themselves as willing to join the Army if their services are needed is experienced in the large towns. In country districts and small towns a much better response is forthcoming, because it is comparatively simple to make arrangements for the protection of a doctor's practice during his absence. His patients are known, and the doctors who remain behind can readily persuade them to be loyal to their usual adviser. Hence on his return he may hope to find his practice substantially unimpaired. But in crowded districts, and especially in London, many patients have little or no attachment to some individual doctor, and a man who is absent for a twelvemonth or more may return to find that a connection which he has established by several years of work has almost disappeared. The risk is a very real one, and it is very difficult by any scheme of organisation fully to meet it. All one can say is that the country is at war, and that individuals are called on to sacrifice themselves in the general interest. The medical profession has certainly shown no failure of patriotism, and it is certain that, whatever the cost, they will not fail now. It is fair to remember that medical officers are accepted on the offer of twelve months' service, while all other military engagements hold until the end of the war.

